# Kinematics of perceived dyadic coordination in dance

**DOI:** 10.1038/s41598-019-52097-6

**Published:** 2019-10-30

**Authors:** Martin Hartmann, Anastasios Mavrolampados, Emma Allingham, Emily Carlson, Birgitta Burger, Petri Toiviainen

**Affiliations:** 1 University of Jyväskylä, Jyväskylä, Finland; 2 University of Hamburg, Hamburg, Germany

**Keywords:** Human behaviour, Computational science

## Abstract

We investigated the relationships between perceptions of similarity and interaction in spontaneously dancing dyads, and movement features extracted using novel computational methods. We hypothesized that dancers’ movements would be perceived as more similar when they exhibited spatially and temporally comparable movement patterns, and as more interactive when they spatially oriented more towards each other. Pairs of dancers were asked to move freely to two musical excerpts while their movements were recorded using optical motion capture. Subsequently, in two separate perceptual experiments we presented stick figure animations of the dyads to observers, who rated degree of interaction and similarity between dancers. Mean perceptual ratings were compared with three different approaches for quantifying coordination: torso orientation, temporal coupling, and spatial coupling. Correlations and partial correlations across dyads were computed between each estimate and the perceptual measures. A systematic exploration showed that torso orientation (dancers facing more towards each other) is a strong predictor of perceived interaction even after controlling for other features, whereas temporal and spatial coupling (dancers moving similarly in space and in time) are better predictors for perceived similarity. Further, our results suggest that similarity is a necessary but not sufficient condition for interaction.

## Introduction

Human ability to coordinate our movements plays a role in dozens of everyday contexts, allowing us to plan and complete motor tasks as simple as walking and as complex as dancing. Coordination has been defined in terms of organizing the degrees of freedom within a motor system in relation to one another^1^; not only does such organization govern our individual movements, but it is also crucial to many interactions with others. Interpersonal coordination has been understood as the “degree to which the behaviors in an interaction are non-random, patterned, or synchronized in both timing and form”^2^. While interpersonal coordination has been studied in a variety of contexts^3^, music-related settings such as joint music performance have particularly provided researchers with many rich examples of complex and precise interpersonal coordination^4^.

When humans dance to music, they are coupling their movements with the music itself, using their body to express musical experience and imitate musical features^5–7^. Although dancing with others is thought to serve many social functions such as group bonding, coalition signaling, and courtship^8,9^, the process of interpersonal coordination in dance has only recently begun to be examined by research. For example, recent studies have investigated relationships between dance movements, the presence of another partner and empathy^10^, as well as perceptions of coordination in dyadic dancing^11^.

Several studies have explored the perceptions of interaction in every-day behaviors such as conversation^12–14^. Research utilizing point-light displays of dyadic interaction has shown that the communicative action of one dyad member allows observers to predict the resulting action of the other dyad member^15^.

It is clear then, that biological motion in human dyadic interaction conveys rich and complex information to observers. However, perception of social interaction in dance movement has not yet been fully explored. Recent research has shown that an individual’s tendency to use empathy (i.e. the ability to interpret and understand others’ thoughts and feelings) relates to their ability to adjust to sudden tempo changes^16^, and to their adjustment of body movement in response to different partners in a dyadic dance context^10^. However, these studies did not measure social perception of movement, and moreover relied on fairly limited analysis of full body movement. Understanding how interaction is perceived in a dance context therefore requires further investigation.

In a recent experimental study^11^, two perceptual dimensions were utilized for the study of interpersonal coordination, namely *interaction* and *similarity*. These two terms have been distinguished in various studies related to *interpersonal coordination*. This notion is typically divided into two main components: *behavioral matching* (or mimicry), which focuses on co-occurrence and imitation of e.g. body posture (the so called ‘chameleon effect’), and *interactional synchrony*, which mainly relates to the pace of communication^17–20^ and to the importance of gaze behavior upon interaction^21^. Based upon this literature, dyadic similarity can be understood as a behavior of more or less delayed matching of movement between dancers, mutual or not, that may involve one or more body parts and movement directions; dyadic interaction would, on the other hand, be more associated with the importance of gaze engagement in order to fall into step with the dyad partner^22^. Further study, however, is required to better understand the relationship between interaction and similarity as components of interpersonal coordination, specifically how this is manifested in dance.

### Quantitative measures of movement coordination

Human motor control studies have proposed the notions of temporal coupling and spatial coupling to describe two distinct and quantifiable types of individual interlimb coordination. Temporal coupling is usually defined as an involuntary convergence between two timing tasks performed by anatomically distinct systems (e.g., speech production and finger tapping), whereas spatial coupling often refers to an involuntary accommodation of shapes of trajectories during motor tasks that require producing different spatial patterns with two limbs^22–24^.

To date, most studies on music-related interpersonal coordination have either utilized a constrained task, such as tapping^25^, or focused on the kinematics of single body parts, such as the head^26^, while kinematic correlates of full-body coordination during unconstrained movement have received less attention. Due to the multidimensionality and non-constrained nature of such coordination, the spatial and temporal modes thereof are likely to vary widely across individuals. Because of this, theory-driven methods for selecting features of whole-body movement to study coupling may provide an incomplete picture, necessitating the use of data-driven methods in understanding the phenomenon of interpersonal coordination in dance. In a study on music-induced movement with single dancers^27^, for example, Principal Component Analysis (PCA) was applied for dimensionality reduction of full-body movement, allowing retention of only the most useful movement components for further analysis.

In contrast to single dancers, to investigate perception of coordination between two dancers it would be obvious to look at temporal covariance (joint variability) between their movements. In the present work we utilize Partial Least Squares Correlation (PLSC)^28^ for this purpose. PLSC, a multivariate statistical technique that was first used in functional neuroimaging^29,30^, can be regarded as a generalization of PCA. While PCA finds uncorrelated linear combinations of the original variables (principal components) that maximize variance within one multidimensional data set, PLSC extracts such linear combinations (PLS components) from two separate datasets so that their covariance is maximized. In the context of movement data, PLSC provides information about both the spatial movement patterns involved in coordination (PLS loadings) and the temporal development thereof (PLS scores).

In addition to temporal and spatial coupling, vision seems to be an important component of interpersonal coordination. For humans and other primates, social interactions rely heavily on complex visual signals including body language and gaze behavior^31–34^. The angle of the head and body likely provide important information about a person’s gaze, especially if the eyes cannot be seen^35^. This suggests that orientation of both the head and the body carry information about where a person is looking, which is an important part of interpreting an interaction.

Taking the above into account, the current study utilizes four computational measures of interpersonal coordination, namely temporal coupling, spatial coupling, torso orientation, and vertical head synchrony. The first two measures relate to temporal covariance of movements and can thus be associated to period locking in dance, whereas torso orientation roughly describes the degree to which both dancers are facing each other. To our knowledge, the temporal coupling and spatial coupling measures described here have not been used before to study interpersonal coordination in music-induced movement. Torso orientation, in contrast, has been previously applied to study the role of trait empathy upon coordination and entrainment^11^. For comparison purposes, we have also computed an additional baseline measure of interpersonal coordination (vertical head synchrony), which focuses on synchrony of vertical movement between the head joints of both dancers.

In this study of interpersonal coordination, temporal coupling and spatial coupling have a different meaning from that in the literature. Instead of referring to intrapersonal coordination phenomena, they involve a representation of the degree of correspondence between each possible pair of body parts and movement directions of two agents. Spatial coupling is an estimate of dyadic coordination that is based on the relevance of each body part and movement direction; two dancers are highly coupled spatially if there is a match in relevance with respect to their corresponding body parts and movement directions.

Torso orientation relates to gaze behavior, which can be mutual or not, so its connection with joint movement imitation is less direct than in the case of the coupling features. Body orientation could be more associated with the idea of a communication or a dialogue between dancers than with mirroring or imitation of movement patterns. In this respect, a strong positive relationship between orientation and perceived interaction has been shown^11^; its relationship with perceived similarity was also positive but did not reach statistical significance.

In the present study we followed up findings from^11^ by investigating prediction of perceived interaction and similarity using computational estimates of movement coordination. In addition, we studied the contribution of novel coordination measures (temporal coupling and spatial coupling) using both data collected in^11^ (Experiment 1) and a new validation data set (Experiment 2). Also, we gained further insights into the perceptual variables investigated in that study (similarity and interaction) by exploring their particular relationship.

In this study, we investigated what types of dance movements were associated with the perception of dyadic interaction and similarity. Dyads were asked to dance freely to music while their movements were recorded using full-body motion capture. Subsequently, in two separate experiments, observers were asked to watch silent stick figure animations of these recordings and to rate interaction and similarity between dancers. Four movement features (temporal coupling, spatial coupling, torso orientation, and vertical head synchrony) were extracted and utilized to quantify coordination. In order to study their prediction ability, these coordination estimates were compared to the perceptual ratings.

The following research questions were addressed in this study:

- What characteristics of dance movement (i.e., temporal coupling, spatial coupling, orientation) are associated with judgments of interaction between dancers?
- What characteristics of dance movement are associated with the perception of similarity between dancers?

We hypothesized that dancers’ movements tend to be perceived as more similar when they exhibit spatially and temporally similar movement patterns, and as more interactive when they spatially orient more towards each other^11,35^. Based upon aforementioned distinctions between similarity and interaction^19,21^, we expected similarity judgements to be mainly associated with spatially congruent and temporally correlated movements between dancers. Interaction, in contrast, would additionally depend more on factors of higher specificity such as the extent to which the dancers were facing towards each other. We highlight that the temporal structure of movement should contribute to perceived interaction as well, especially so for leader-follower relationships^36^ and patterns of turn-taking (which are distinct from unison or imitation^37^).

**Figure 1:**
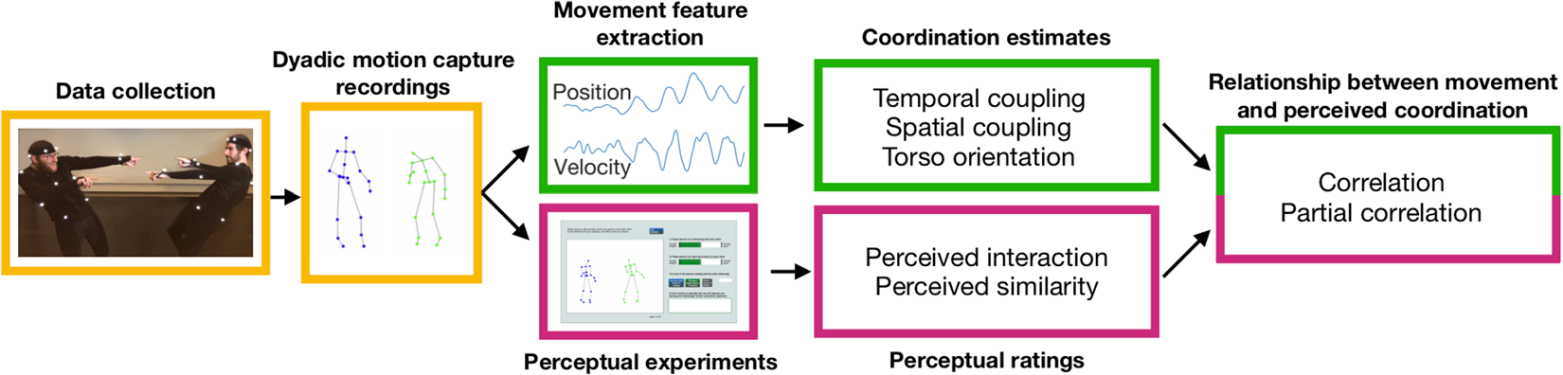
General design and workflow of the study.

Two perceptual experiments were conducted in this study, based on dyadic motion capture recordings (20 s in length). In Experiment 1, 24 stick figure animations of dyads dancing to music were shown to 33 raters. Experiment 2 used larger sample sizes: 35 animations of dyads were shown to 50 raters. In both perceptual experiments, participants were asked to rate the degree of interaction and similarity between dancers. Coordination estimates were extracted from the movement data and correlated, across dyads, with the perceptual ratings of interaction and similarity. Figure 1 shows the general design of the study.

## Results

The perceptual ratings were tested for possible outliers with mean inter-subject correlations. Correlations between each possible pair of participants across stimuli ratings were computed and averaged across participants to estimate how much consensus there is between each rater and the rest. Participants with negative correlations in either interaction or similarity were first excluded. Following this, participants with correlations of more than two standard deviations from the overall mean inter-subject correlation across participants were also removed. In Experiment 1, this resulted in the exclusion of two participants for both interaction and similarity. In Experiment 2, two participants were excluded for interaction and three for similarity. Subsequently, Cronbach’s alpha showed high consistency for both ratings scales and experiments, suggesting that there was an overall agreement among raters (Table 1). The ratings of each dyad were then averaged across participants to provide a mean rated interaction and similarity score.

**Table 1:** Mean inter-subject correlations and Cronbach’s alpha for perceptual ratings.

Statistic	Experiment 1		Experiment 2	
	Interaction	Similarity	Interaction	Similarity
Mean Inter-subject Correlation	0.57	0.39	0.53	0.45
Cronbach’s alpha	0.86	0.76	0.85	0.87
*N*	31	31	48	47
*M*	46.02	57.26	47.14	53.08
*SD*	24.7	18.52	19.05	15.9

Independent samples *t*-tests were run on the perceptual ratings of the two musical stimuli to check whether they had an effect on the raters’ judgements. There were no significant differences between the two musical stimuli in any of the perceptual ratings for both experiments. Independent samples *t*-tests were also run on the rated interaction and similarity scores between the two experiments. There were no significant differences between the two experiments in either perceptual rating, suggesting that interaction and similarity ratings in Experiment 1 were comparable to those in Experiment 2. In addition, similarity received higher ratings than interaction and a paired samples *t*-test between the two scales was significant for Experiment 1, *t*(22) = 2.69, *p* < 0.05, and Experiment 2, *t*(33) = 2.55, *p* < 0.05.

**Figure 2:**
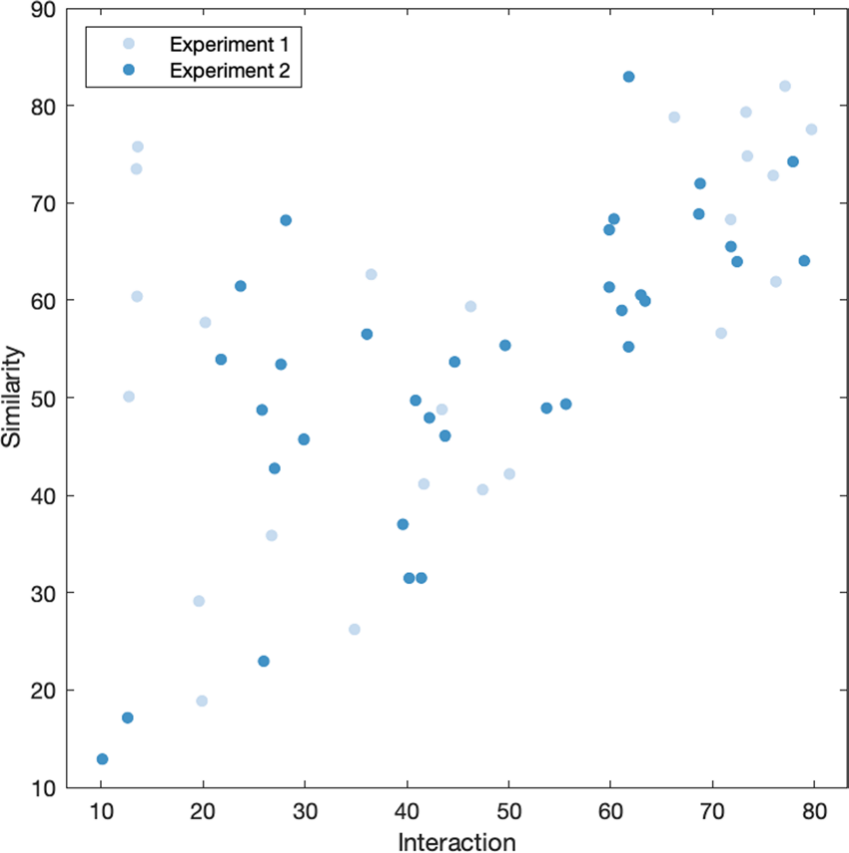
Relationship between interaction and similarity ratings.

Rated interaction and similarity were positively correlated in Experiment 1 (*r*(22) = 0.52, *p* < 0.05) and Experiment 2 (*r*(33) = 0.70, *p* < 0.001). The scatter plot in Fig. 2 shows that high similarity ratings are associated with low interaction ratings, but not the other way around. To measure the interaction between perceptual variables in the upper left part of the scatter plot, we obtained the skewness of the difference between interaction and similarity for Experiment 1 (*b* = −1.08) and for Experiment 2 (*b* = −0.92). The scatter plot and negative skewness in both experiments suggest that, in addition to the observed linear relationship between variables, a proportion of the dyads exhibits high perceived similarity and low perceived interaction.

### Collinearity between coordination estimates

To check for collinearity between coordination estimates, Pearson correlations with two-tailed significance testing were calculated between the coordination estimates (Table 2 for Experiment 1 and Table 3 for Experiment 2). There was a significant correlation only in Experiment 1 between temporal coupling and torso orientation (*r*(22) = 0.48, *p* < 0.05), suggesting that the correlations were overall low and the three estimates described different aspects of movement.

**Table 2:** Correlations between coordination estimates (Experiment 1).

Coordination Estimate	Temporal Coupling	Spatial Coupling	Torso Orientation
Spatial Coupling	0.28	—	0.20
Torso Orientation	0.48*	0.20	—
Vertical Head Synchrony	0.36	0.06	0.33

*p < 0.05, two-tailed.

**Table 3:** Correlations between coordination estimates (Experiment 2).

Coordination Estimate	Temporal Coupling	Spatial Coupling	Torso Orientation
Spatial Coupling	0.24	—	0.18
Torso Orientation	0.18	0.18	—
Vertical Head Synchrony	0.19	0.28	0.21

### Correlation between coordination estimates and perceptual ratings

**Figure 3:**
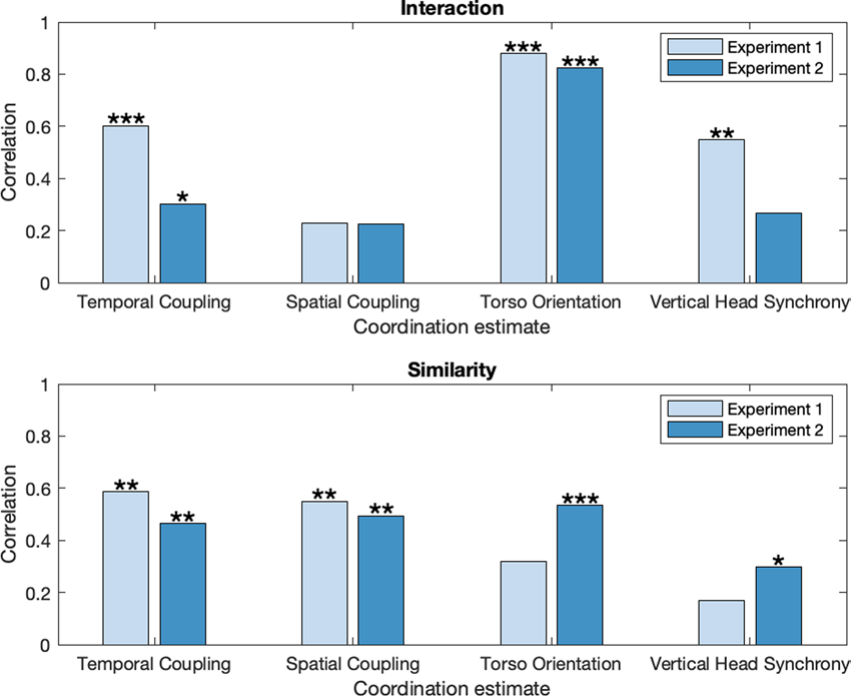
Correlations between coordination estimates and perceived interaction as well as perceived similarity (*p < 0.05; **p < 0.01; ***p < 0.001).

Each coordination estimate was correlated with the mean interaction and similarity ratings using one-tailed tests. Figure 3 shows correlation effect sizes and significance levels for each experiment. To provide a single estimation of the significance of each measure, the p-values of the correlations across the two experiments were pooled; first, the p-values from the individual experiments were converted to z-scores with the standard normal cumulative distribution function, then the thus obtained z-scores were combined to an overall z-score using Stouffer’s z-score method^38^. In the following, we focus mainly on significance obtained from pooled estimates. Table 4 presents pooled z-statistics of the correlations between perceptual judgements and coordination estimates. All four coordination estimates were significantly correlated with both perceived interaction and similarity, although there were differences in the effect sizes and significance levels. Torso orientation and temporal coupling were significantly correlated with rated interaction (pooled *p* < 0.001), with torso orientation having the highest effect sizes in both experiments. Rated similarity was significantly correlated with temporal coupling, spatial coupling and torso orientation (pooled *p* < 0.001), and with vertical head synchrony (pooled *p* < 0.05).

**Table 4:** Pooled z-scores from correlation between coordination estimates and perceptual measures.

Coordination Estimate	Interaction	Similarity
Temporal Coupling	3.45***	4.12***
Spatial Coupling	1.67*	4.10***
Torso Orientation	8.28***	3.41***
Vertical Head Synchrony	3.06**	1.78*

*p < 0.05; **p < 0.01; ***p < 0.001.

### Partial correlation between coordination estimates and perceptual ratings

**Figure 4:**
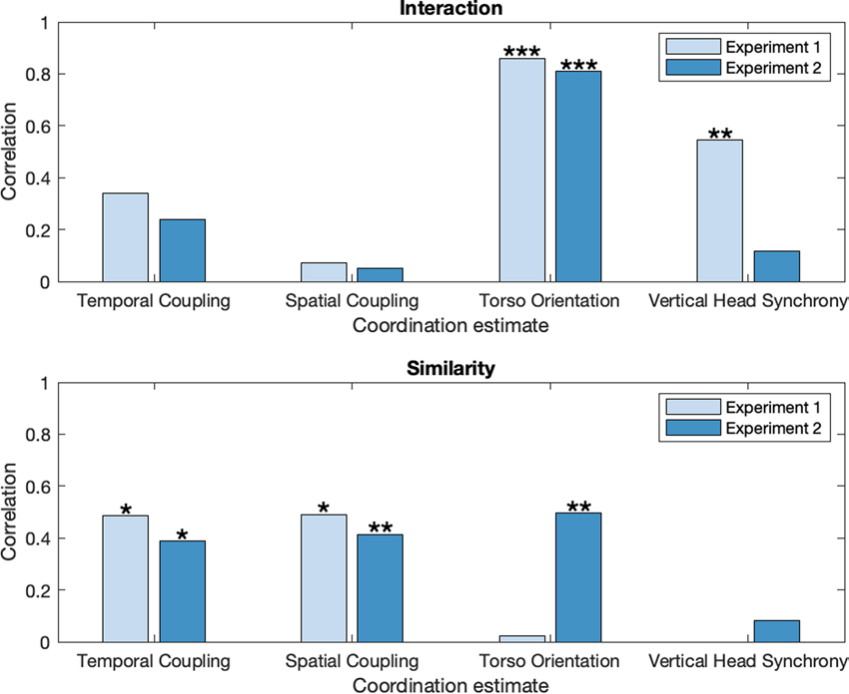
Partial correlations between coordination estimates and perceived interaction as well as perceived similarity (*p < 0.05; **p < 0.01; ***p < 0.001).

Partial correlations were conducted to identify the unique contribution of each coordination estimate on the perceptual ratings. Figure 4 presents effect sizes and one-tailed significance levels of partial correlations, while their respective pooled z-statistics are shown in Table 5. These analyses revealed that for interaction, torso orientation was the most highly partially correlated estimate (Experiment 1 *r*(22) = 0.84, Experiment 2 *r*(33) = 0.81, pooled *p* < 0.001). Temporal coupling and vertical head synchrony were also significantly partially correlated with rated interaction (pooled *p* < 0.05), but had small effect sizes overall. In contrast, partial correlations between spatial coupling and rated interaction did not reach statistical significance. As regards rated similarity, statistically significant partial correlations were found with spatial coupling and temporal coupling (pooled *p* < 0.001), as well as torso orientation (pooled *p* < 0.05), with all three estimates having moderate effect sizes. Finally, the partial correlation between rated similarity and vertical head synchrony did not reach statistical significance.

**Table 5:** Pooled z-scores from partial correlation between coordination estimates and perceptual measures.

Coordination Estimate	Interaction	Similarity
Temporal Coupling	2.00*	3.13***
Spatial Coupling	0.41	3.26***
Torso Orientation	7.50***	2.11*
Vertical Head Synchrony	2.25*	0.20

*p < 0.05; **p < 0.01; ***p < 0.001.

## Discussion

The current study aimed to expand findings on kinematic correlates of perceived coordination in unconstrained, improvised dance movement. A full-body approach was chosen to account for the multidimensionality of human movement, employing a set of novel computational coordination estimates and perceptual measures. Two perceptual experiments with sample groups of distinct demographics were conducted to validate the computational measures in different populations. Overall, results were similar for the two experiments, suggesting that the effects of the coordination estimates on the perceptual ratings could be generalised across the two samples. A few noticeable differences between experiments were found, which are discussed in this section.

Based upon previous work distinguishing similarity and interaction, we hypothesized that dyads whose dancers shared resemblances with each other due to covarying local or global movements may be perceived as being more similar. Interaction, on the other hand, was hypothesized to be better associated with a dynamic dialogue having some degree of turn-taking organization, which would also involve coupling but rely heavily on gaze for its regular development. Our results lend general support to these hypotheses, particularly because they show the contribution of torso orientation upon perceived interaction and the role of temporal and spatial coupling on perceived similarity.

The crucial importance of orientation upon interaction has been highlighted in the literature. For instance, work on nonverbal behavior in ‘free running’ dyadic interaction showed several relationships between gaze and interaction^34^. Our study found that torso orientation also has a role on similarity prediction, which has been previously observed in work on unintentional interpersonal coordination^39^.

It is interesting to note that the data-driven PLS methods utilized here to analyze full-body coordination (temporal coupling and spatial coupling) were, overall, more accurate for the prediction of perceived similarity, whereas torso orientation and vertical head synchrony, which focus on specific body parts or movement directions, yielded higher prediction accuracy for interaction. In this respect, it has been previously shown^12^ that theory-driven measures focusing only on few body parts (i.e., amount of hand movement and autocorrelation-based period locking) were also more accurate at predicting interaction than similarity.

### Perceived interaction

The results regarding perceived interaction were consistent with our hypotheses, because more frontal orientation between dyad members led to significantly higher interaction ratings. In addition, vertical head synchrony and temporal coupling also exhibited positive correlations but with smaller effect sizes. In contrast, spatial coupling failed to show any linear relationship with perceived interaction when other coordination measures were controlled for.

The clearest result of the study was the observed correlation between perceived interaction and torso orientation, which remained high even after controlling for the other coordination measures. Assuming that torso orientation describes, at least to some extent, eye or face gaze behavior, this result resonates with findings showing the importance of visual information upon interactional synchrony^21^. Considering that proportion of looking can be a strong predictor of perceived interaction, our results would suggest that behaviors such as assumed eye contact (or mutual gaze) and staring (continual gaze at the other dyad member) whilst dancing in dyads are associated with higher perceived interaction, whereas one-sided gaze (one of the dyad members does not return gaze) and gaze aversion (looking away) should yield lower ratings of perceived interaction (see^40^ for a distinction between gaze variables).

A statistically significant relationship between perceived interaction and temporal coupling was observed based upon correlations and partial correlations, although the effect sizes were lower than for torso orientation. This suggests that in-phase synchronization of beats or rhythms between partners contributes, at least to some extent, to the perception of interaction, a finding that is consistent with the literature on interactional synchrony^19^. A caveat regarding the partial correlations is that only the pooled analysis yielded statistically significant results.

### Perceived similarity

In agreement with our expectations, spatial coupling and temporal coupling exhibited statistically significant correlations with judgements of perceived similarity. Also, torso orientation exhibited positive correlations, but with lower effect sizes for the first experiment. The baseline measure of vertical head synchrony, in contrast, only exhibited weak or no partial correlation with perceived similarity.

According to the partial correlations with perceived similarity, spatial coupling exhibits the largest overall effect size (Fig. 4) and significance (Table 5), closely followed by temporal coupling. In contrast, spatial coupling yielded very low partial correlations for perceived interaction, suggesting that this feature could help to understand the discrepancies between perceived similarity and interaction. It is plausible, in this respect, to associate perceived similarity with behavioral mimicry or behavioral matching, which refers to similarity in form (e.g., body posture) between subjects within a temporal window spanning up to about 10 seconds^19,21^. However, similarity exhibited high partial correlations not only with spatial coupling but also with temporal coupling and torso orientation. This finding could suggest that perceived similarity has more underlying dimensions and describes a phenomenon of greater complexity than interaction. It seems that temporal synchrony and visual contact, which are variables that have typically been connected with interactional synchrony^21^, could also be considered as correlates of perceived similarity. Overall, these findings suggest that perceived similarity could be associated with mirroring behavior during dancing. Our findings indicate the possible contribution of three factors upon perceived similarity, which are operationalized by spatial coupling, temporal coupling, and torso orientation, respectively: spatial match with respect to body parts and movement directions that are most representative of the joint movement, movement timing coordination (i.e., period and phase locking between same or different body parts and movement directions), and some degree of visual contact to reproduce each other’s movements.

The significant correlations between similarity and torso orientation warrant further explanation. Its pooled significance was lower than for the coupling measures, but this reflects differences between experiments in the obtained correlations and partial correlations. Torso orientation was correlated significantly with similarity in Experiment 2, whereas there was no effect for Experiment 1. This finding does not seem to arise from differences in torso orientation variance between experiments, as the standard deviation across participants for maximum torso orientation across windows was similar in both experiments (Experiment 1 *SD* = 0.72, Experiment 2 *SD* = 0.70). It is worth noticing, however, that perceived similarity yielded overall lower mean inter-subject correlations and Cronbach’s alpha than perceived interaction, especially for Experiment 1 (Table 1), so the similarity results for the first experiment should be treated with more caution. On a more general note, this lower consensus for perceived similarity ratings is compatible with the aforementioned interpretation that perceived similarity may describe a more complex phenomenon than perceived interaction.

### Relationship between perceived similarity and interaction

We conducted an exploratory analysis regarding the differences in prediction accuracy for interaction and similarity, and the nonlinear relationship between these two variables in both experiments. As previously shown in Fig. 2, high perceived similarity and low perceived interaction is observed for a proportion of the dyads. On the other hand, dyads with high perceived interaction always exhibit high perceived similarity. This interesting relationship between similarity and interaction suggests that similarity is a necessary condition for interaction, but interaction may not be a necessary condition for similarity. In other words, high interaction ratings are always associated with high similarity ratings, but high similarity ratings may or may not be associated with high interaction ratings. On a more general note, these results, which warrant further investigation, show that interaction and similarity describe different aspects of coordination in dance. The supplementary video to this article includes examples of dyads that were selected based on their similarity and interaction ratings; dyads that were rated high in similarity and low in interaction can be found among these examples.

The particular relationship between interaction and similarity could suggest that both variables can be associated to performing similar movements, e.g. as a response to the musical beat; however, it seems that facing towards each other is a necessary condition for observers to rate higher interaction between dancers. In this sense, interaction can be understood as a special case of similarity in which mutual orientation is present. Related to this, our findings somewhat resonate with the dichotomy proposed in previous studies between interactional synchrony and behavioral mimicry^19,21^, suggesting that perceived interaction could be related to gaze behavior whilst dancing, whereas perceived similarity between dancers could be more associated to spatiotemporal mirroring behavior.

## Conclusions

This study explored perceptions of interaction and similarity in dance dyads, and their relationship to computationally extracted movement features. Our findings showed that the two perceptual variables exhibited different profiles in their correlation with the coordination estimates, suggesting that they describe distinct constructs. Perceived interaction was mainly related to the extent to which the dancers were facing each other, with torso orientation being the best predictor and having large effect sizes in both experiments; vertical head synchrony and temporal coupling also seemed to predict perceived interaction but these results were less consistent and of lower statistical significance. On the other hand, perceived similarity correlated significantly with all the three proposed estimates, with spatial coupling yielding the highest predictive accuracy across datasets, followed by temporal coupling.

Finally, we suggest some possible directions for future research in interpersonal coordination, focusing on both operationalization and broader methodological considerations. Future work could go beyond our investigation of similarity and interaction by including other possibly related perceptual dimensions, such as ‘synchronization’ or ‘togetherness’^41^. It would be interesting to gain new insights regarding their possible commonalities and to study how they differ with respect to predictive accuracy from computational synchrony estimates.

Our findings could be further investigated through future controlled studies in which some of the key factors of interest would be manipulated via instructions to dancers. For instance, different dyad members or groups of dyads could be asked not to interact (or e.g. not to face each other), to moderately interact, or to fully interact. Additionally, motion capture recordings could be manipulated by introducing a phase delay or a tempo difference between the movements of dyad members, in order to investigate whether reduced phase and period locking leads to lower perceptual ratings. Also orientation manipulation, e.g. rotating one of the dancers, would allow to examine possible differences between mutual and non-mutual gaze upon interaction.

An interesting avenue of research that further studies could tackle involves understanding the effects of synchronization with the music upon interpersonal coordination. It is likely that the observed interpersonal coordination was induced both by visual cues given by the dancers to one another as well as by the musical stimuli. In this sense, *social entrainment* has been defined as a special case of spatiotemporal coordination where the rhythmic signal comes from another individual^42^. While our approach does not specifically address the relative contributions of social entrainment and the dancers’ spatiotemporal coordination to the musical cue, the relationship between perceived similarity and perceived interaction could relate to the presence of a coordinating audio signal in addition to the visual signal of the dance partners’ movements. It would be interesting to explore the relative contribution of intrapersonal auditory phenomena and of mutual social entrainment to perceived coordination. This problem has been investigated using a rocking chair paradigm^43^ and should also be studied in the context of unconstrained dance movement. A possibility would be to conduct a modified version of the perceptual experiments reported in this study, which would be based on *pseudo interactions*^44–46^, i.e., the dyad members would seemingly dance together but their movements would actually come from different recordings done with other partners.

In relation to this, our aim was to tackle the problem of movement coordination from a concurrent viewpoint. An interesting approach for further studies would be to focus on non-concurrent estimation of coordination, exploring possible delays between dancers and recurrence of their movement patterns. These methods include: (I) applying time shifting to the data used for PLS computation; (II) using multivariate recurrence quantification analysis^47^; (III) estimation of mutual information.

Finally, further work is needed to test the validity of these findings using a more representative sample. It would be important to recruit participants of different dancing and cultural background for both motion capture and perceptual experiments. For example, skilled dancers may still be perceived as interacting with each other regardless of their body orientation. Other scenarios that could provide new insights for interpersonal coordination in music-induced movement include children’s dance and normative (teacher-student) dance training.

## Methods

### Ethical approval and informed consent

The motion capture study and the perceptual experiments described below took place at the Department of Music, Art and Culture studies of the University of Jyväskylä. All experiments were performed in accordance to the guidelines and regulations of the National Advisory Board on Research Ethics in Finland (TENK, see https://www.tenk.fi/sites/tenk.fi/files/ethicalprinciples.pdf) relating to research in the humanities and social and behavioral sciences, which the University of Jyväskylä Ethical Committee adheres to. Ethical permission was not needed for this kind of research, according to the aforementioned guidelines and regulations.

Participation in the motion capture study and the perceptual experiments was completely voluntary and was organized and supervised by authors of the study. The key experimental procedures were explained to participants in advance, who gave their written consent for participation and further use of the collected research data in this research project and potential follow-ups. Participants were also informed that they could withdraw from the research at any time. They were informed of the general purpose of the motion capture study and the perceptual experiments, but they were not informed at any point about the research hypotheses. Participants were debriefed as to study objectives prior to their departure.

### Motion capture study

The motion capture study was designed to collect free dance movement data from participants in a dyadic setting, using naturalistic (commercially available) stimuli.

#### Participants

Participants were recruited using social media and University e-mail lists. Seventy-three (52 female) participants, aged 19–40 years (M = 25.75, SD = 4.72) completed the motion capture experiment. Participants were of 24 different nationalities and received two movie ticket vouchers in exchange for their participation.

#### Apparatus

**Figure 5:**
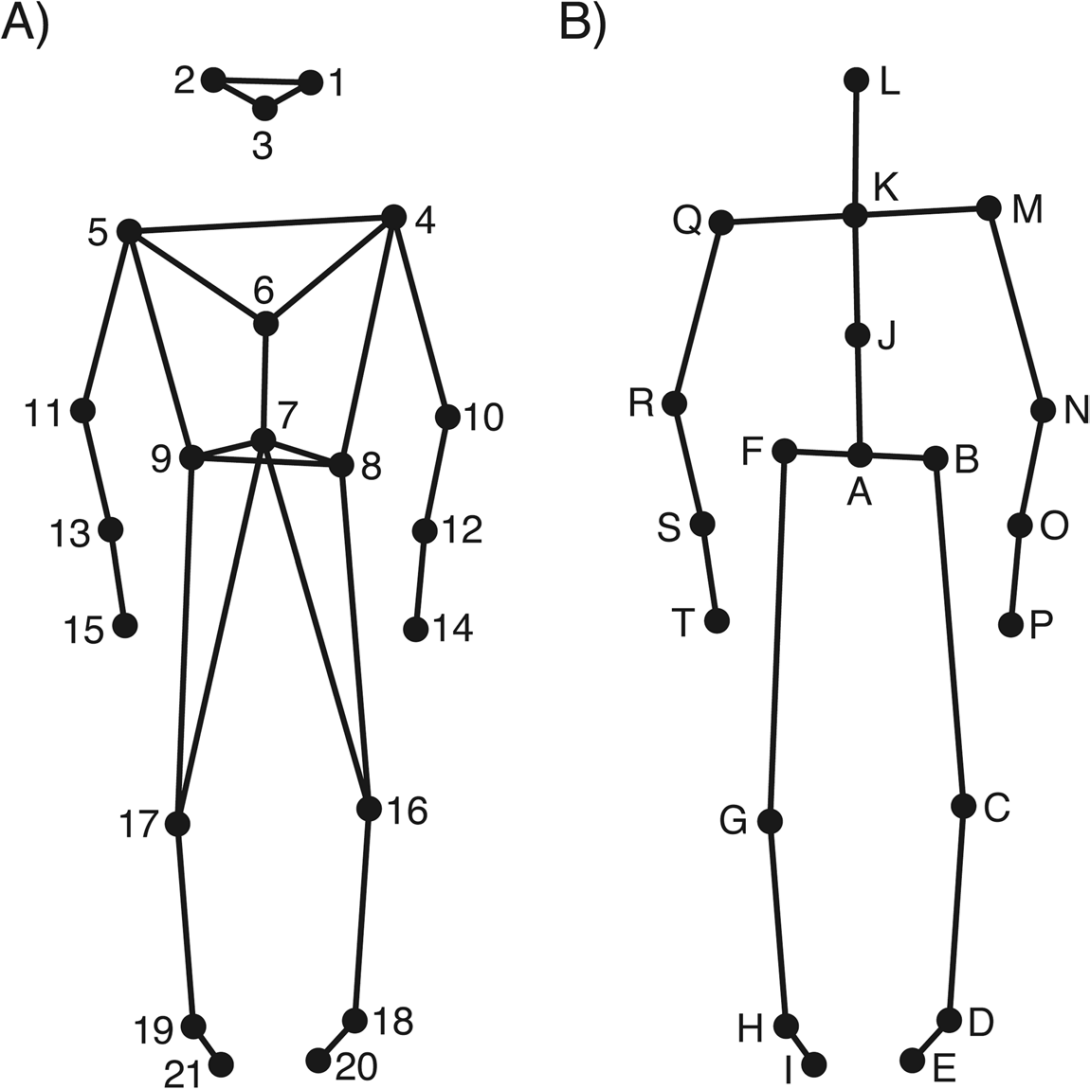
Stick figure illustrations of marker and joint locations. (**A**) Anterior view of the original marker locations; (**B**) Anterior view of the locations of the reduced secondary markers/joints used in animation and analysis of the data.

Participants’ movements were recorded using a twelve-camera optical motion capture system (Qualisys Oqus 5+), tracking the three-dimensional positions of 21 reflective body markers attached to each participant, at a frame rate of 120 Hz. The locations of markers are shown in Fig. 5A. The musical stimuli were played in a random order in each condition via four Genelec 8030 A loudspeakers and a sub-woofer. The direct (line-in) audio signal of the playback and the synchronization pulse transmitted by the Qualisys cameras when recording were recorded using ProTools software so as to synchronize the motion capture data with the musical stimuli afterwards.

#### Procedure

Participants attended the experiment in groups of three or four and were instructed to move freely to the randomized musical stimuli, as they might in a dance club or party setting. The stimuli comprised 16 excerpts from 8 genres: Blues, Country, Dance, Jazz, Metal, Pop, Rap and Reggae. Stimuli for the experiment were selected computationally using social tagging to identify excerpts most representative of the chosen genres (see^48^ for a full description and discussion of this process), and ranged from 118–132 BPM. Participants first danced individually (this data was not used for this study), and then danced in each possible dyad combination, yielding six dyad combinations for groups of four, and three dyad combinations for groups of three; a screen divider was used to split the room so that two different dyads could be recorded at the same time. The total number of dyads recorded was 99.

#### Stimuli processing and animation

Using the Motion Capture (MoCap) Toolbox^49^ in MATLAB, movement data of the 42 markers (21 per dyad member) were trimmed to match the exact duration of the musical excerpts and gaps in the data were linearly filled. Data were then trimmed a second time to comprise a range of the 5th through the 25th second of the recording from the start of the music, resulting in excerpts of 20 seconds each. Following this, the data were transformed into a set of 40 secondary markers, subsequently referred to as joints, of which there were 20 per dyad member. The locations of these 20 joints are depicted in Fig. 5B.

Following the motion capture data preprocessing, data were animated at a rate of 30 frames per second and were rendered in color such that one dyad member was animated in blue and the other in green. To avoid the possibility that the position of the dancer on the left or right would affect participants’ perceptions, two sets of data were created such that in one set, the green dancer was positioned on the left while the blue dancer was positioned on the right; in the second set, these were swapped. The viewing azimuth angle of each animation was subjectively chosen so that the movement of both dancers was clear (i.e the figures were not overlapping), and the view was even for both dancers (i.e one dancer did not appear closer to the camera than the other). Animations were created without audio so that observers would focus on coordination between dancers rather than on synchronization to the music.

### Perceptual experiments

**Figure 6:**
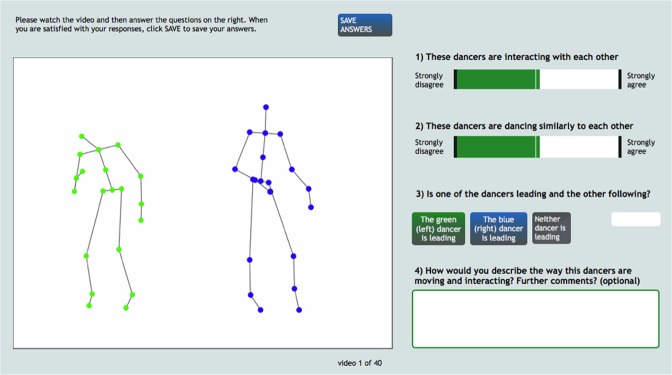
Perceptual study self-guided interface.

The animations of motion capture data were used to create a perceptual experiment exploring visual perceptions of interpersonal coordination during dance (see Fig. 6). This perceptual experiment was run twice, using two separate samples of stimuli and observers. A detailed description of the procedure and creation of stick figure animations can be found in^11^.

#### Stimulus selection

A total of 24 dyad recordings were presented in Experiment 1, whereas 40 recordings were shown in Experiment 2. For the first perceptual experiment, 12 female dyads (mean age 26, SD = 4.3) were selected based on the dancers’ self-reported trait empathy, measured using the Empathy Quotient (EQ)^50^. Dyads comprised of dancers whose empathy scores fell into the top or bottom quartiles of all empathy scores were selected, with the aim of choosing only those with extreme empathy scores. This process allowed the investigation of the role of empathy in dyadic coordination^11^. This sample of dancers represented a range of nationalities (15) with Finnish as the most represented. For the second perceptual experiment, 20 new dyads (34 female, mean age 25, SD = 4.5) were selected. For this sample, to limit potential effects of culture, 35 same-gendered dyads whose members were European or North American were chosen.

For both perceptual experiments, recordings of the selected dyads dancing to two pop music stimuli were used. Therefore, both experiments dealt with the same musical stimuli, which was limited to pop music. This genre was chosen based on dancers’ preference for pop music, and generally high variability of overall amount of movement (see^11^ for a detailed description of this selection process). This sample of dancers represented a variety of nationalities (15), but the most represented nationality was Finnish.

#### Participants

Participants for the perceptual experiments were recruited through student mailing lists at the University of Jyväskylä, social media advertising, and flyers around the university campus. After the experiment, participants received a movie ticket voucher in exchange for their participation in the study. Any applicants who had taken part in the motion capture study described above were excluded from participating in the perceptual experiments.

Perceptual experiment 1: A total of 33 (25 female) participants took part, with an age range of 23 to 56 (M = 30.1, SD = 7.2). Mean years of musical training was 3.8 (SD = 6.2), and mean years of dance training was 1.6 (SD = 3.5). The sample represented a wide range of nationalities (19), with the most represented nationality being Finnish.

Perceptual experiment 2: A total of 50 participants (36 female) took part, with an age range of 21 to 54 (M = 27.46, SD = 6.19). Mean years of musical training was 5.58 (SD = 6.43), and mean years of dance training was 2.26 (SD = 3.92). The sample represented various nationalities (12), with Finnish as the most represented.

### Kinematic feature extraction

We developed novel computational methods for extracting coordination estimates related to temporal, spatial and orientation aspects of movement. Motion capture data used to create the perceptual stimuli in each of the experiments were further analyzed using the MoCap Toolbox^49^. Coordination estimates from the 24 stimuli shown to participants in Experiment 1 and from the 35 stimuli used in Experiment 2 were computed. Subsequently, markers were transformed to joints (20 per dyad member) and missing motion capture data were filled.

#### Coordination measures

Three window-based coordination estimates (*torso orientation*, *temporal coupling*, and *spatial coupling*) and a baseline measure (*vertical head synchrony*) were extracted either from position data or velocity of dancers. Velocity data were extracted from the vertical axis of the head joint by computing the difference quotient between successive frames and applying a second-order low pass Butterworth smoothing filter with cut-off frequency of 12 Hz. As the movements of the dancers were non-stationary due to the free nature of the task, position and velocity times-series of each dancer were split into windows of shorter duration, allowing for a dynamic approach. After testing various parameters of the moving window, we found the optimal window length to be 10 s (half of the stimuli duration). Therefore, for all coordination estimates, moving windows of 10 seconds with a hop factor of one second were applied to the data of each dancer.

Vertical head synchrony: A baseline measure focusing on vertical synchrony of the head joint and often employed in existing literature^51^ served as a comparison to our model. For each window, the velocity data of the vertical axis of the head joint were correlated between the dyad members.

**Figure 7:**
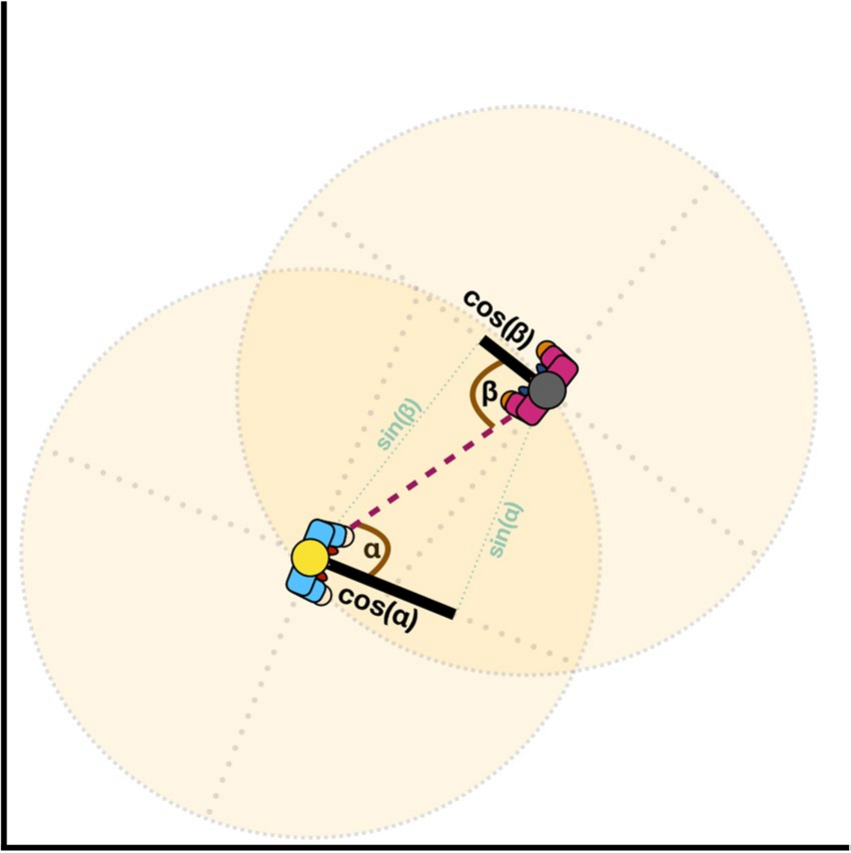
Illustration of the torso orientation measure.

Torso orientation: We computed a quantitative measure to estimate the degree to which the dancers were facing each other. This estimate was derived from position data of the dyad members, and is illustrated in Fig. 7 with two dancers located in a two-dimensional space at a certain point in time. A vector orthogonal to the line joining the shoulders was projected anteriorly to the body, as an estimation of the instantaneous direction the dancer was facing. The cosine of the angle between that vector and the mean position of the shoulders and torso of the other dancer (shown as a dashed segment) was computed as a function of time for each dancer. The cosine values of the dyad members were then summed together, yielding an estimate for each timepoint. Subsequently, the temporal mean was calculated, resulting in a single orientation estimation per window and dyad. This measure is referred to in the analysis as torso orientation.

For the subsequent analyses we transformed the data of each dancer into a local coordinate system in which the mediolateral axis was defined to be parallel to the line joining the mean locations of the hip markers (F and B). Three-dimensional velocity data were extracted from each of the 20 joints. This resulted in a 60-dimensional velocity time-series (20 joints by 3 movement directions) for each dancer.

**Figure 8:**
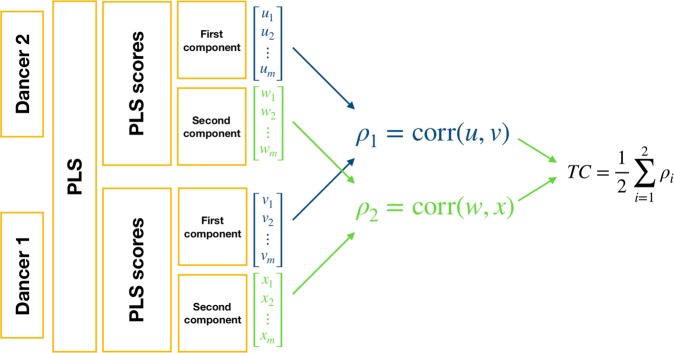
Illustration of the steps involved in the computation of the temporal coupling (TC) estimate for each temporal window.

Temporal coupling: To obtain an estimate of temporal coupling between dancers we utilized the PLSC method. In keeping with standard PLSC procedure^28,29,52^, a 60 × 60 covariance matrix was obtained from the data of each dancer, per window and dyad. Each row (or column) of the covariance matrix is a 60-dimensional vector that corresponds to one of the joints, and whose magnitude indicates its relationship to other body parts and movement directions. Subsequently, singular value decomposition (SVD) was performed on the covariance matrix. Briefly, SVD is a method that factorizes a matrix into the product of three matrices; a diagonal matrix where singular values are entered as diagonal elements, and two matrices with orthogonal columns (often referred to as left and right singular vectors). The orthogonal left and right singular vectors correspond respectively to each dyad member and contain the coefficients (PLS loadings) of each component to the original variables (joints). The diagonal matrix of singular values (comparable to eigenvalues in PCA) contains information about the amount of covariance explained by each component. PLS scores were obtained by matrix multiplication between the mean-centered velocity data and the PLS loadings, resulting in one-dimensional time-series scores per component and dyad member. The first two PLS components were selected for subsequent analyses as they yielded optimal results. To assess the degree of coordination, Pearson’s correlation was applied to the corresponding PLS scores of the dyad members for each component and window. The mean correlation coefficient across components was then taken, resulting in a single estimation per window and dyad. This measure, illustrated in Fig. 8, captures the temporal similarity of the PLS components between the two dancers and will be referred to as temporal coupling in the subsequent analyses. An interesting aspect about temporal coupling is that the body parts and movement directions that contribute to the estimate can be completely different for each of the two dancers, and are chosen based on the covariance matrix, i.e., on how well they represent the body movements of both dancers.

**Figure 9:**
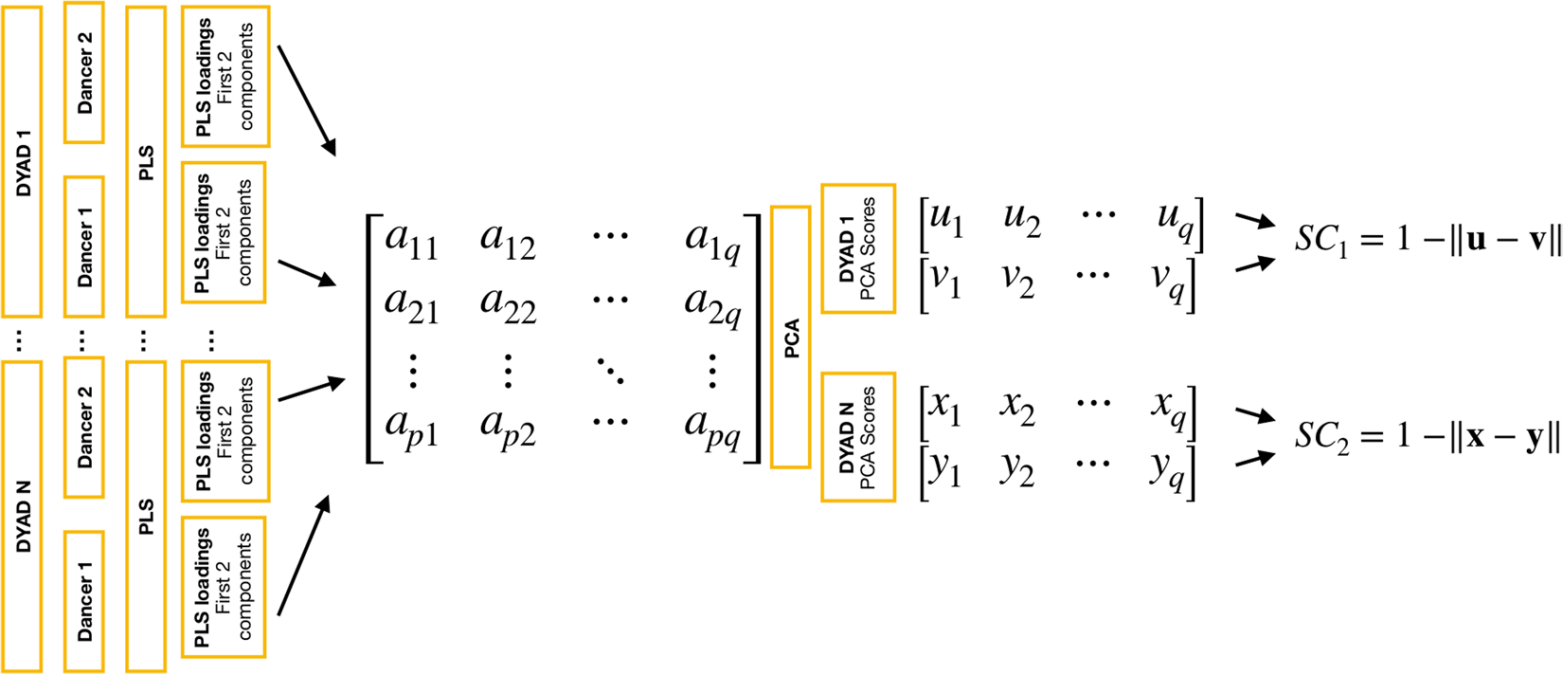
Illustration of the main steps involved in the calculation of the spatial coupling (SC) estimate for each temporal window.

Spatial coupling: The covariance matrix used in PLS is computed from all possible pairs of joints and movement directions between dyad members, resulting in PLS components that can represent coordination originating from different body parts/movement directions for each dancer (e.g., hands for dancer 1 and toes for dancer 2). However, PLS components originating from the same body parts/movement directions in each dancer might be perceptually more salient to the raters. For this reason, a measure of the similarity between coordinated spatial movement patterns of the two dancers was devised. Principal Component Analysis (PCA) was applied to PLS loadings across all windows and dyads to reduce dimensionality and to identify the main modes of coordination across all dancers. Similarity between dancers was calculated with the Euclidean distance between the absolute values of the PC scores of the two dancers for each window and dyad. By taking the absolute values, we ignore whether the movements have in-phase or anti-phase relationship. This measure, based on similarity of the PC projections of the loading vectors, is referred to as spatial coupling, and the main steps of its computation are illustrated in Fig. 9.

#### Correlation between computational coordination estimates and perceptual ratings

To provide a single score for each coordination estimate across the windows of each dyad, we selected the dyad window with the maximum score as it yielded optimal results over other approaches (namely, mean and standard deviation across window scores). The resulting scores of each coordination estimate were correlated with the respective perceptual ratings of similarity and interaction for both experiments. ‘Coordination estimate’ is subsequently referred to as the maximum score across windows. Partial correlations were carried out for each estimate separately in order to control for the other three coordination estimates and to identify the unique contribution of each measure to the perceptual ratings.

## Supplementary information

Supplementary video

## Data Availability

The authors are fully commited to make materials (e.g, stick figure animations), data (e.g., extracted movement features) and associated protocols (e.g., perceptual study interface for data collection, code used for feature extraction and data analysis) promptly available to readers without undue qualifications.
